# Citation Analysis of Papers of Research Projects at Faculty of Dentistry, Tabriz University of Medical Sciences

**DOI:** 10.5681/joddd.2011.025

**Published:** 2011-09-05

**Authors:** Sima Esmaeilzadeh Hojbi, Saeed Ghaffari

**Affiliations:** ^1^Librarian in Charge of Library, Faculty of Dentistry, Tabriz University of Medical Sciences, Tabriz, Iran; ^2^Master Student, Department of Library and Information Sciences, Faculty of Human Sciences, Islamic Azad University of Hamedan, Hamedan, Iran; ^3^Assistant Professor, Department of Library Science and Information, Qom Payame Noor University, Qom, Iran

We attempted to evaluate the researchers’ behavior in using scientific resources in the Faculty of Dentistry at Tabriz University of Medical Sciences. The importance of such study is highlighted when financial resources are limited and the scholarly publications are on an ever-increasing rate, necessitating the selection of the most efficient resources for the libraries. In Iran, 80.5% of the university central libraries depend on the policies of Ministry of Health and Medical Education for subscription to periodical publications on medical sciences.^1^ Since libraries serve as connection routes between previous knowledge and new information, their organizational needs should be analyzed by means of scientific methods as well as quantitative and statistical approaches.^2^

We evaluated the references of articles published as a derivative of approved research proposals in Faculty of Dentistry, Tabriz University of Medical Sciences during a five-year period (2005-2010) using scientometric methods. Only published journal articles present in the faculty member records were included. Departments of Oral and Maxillofacial Radiology and Community Dentistry did not have papers that matched inclusion criteria. 74 papers with a total of 1723 references (mean number of references: 23.28) from nine departments were included (Table 1). Most references were journal articles (93.7%) and in English (98.3%).

Half life formula was used for determining the median age of articles cited.^3^ Journal half life was 10.44 years, indicating more than 50% of the cited articles were of recent ten years. The half life of the books was 7.17 years, indicating that more than 50% of cited books were of recent seven years.

In order to determine core journals, Bradford method was applied.^4^ In total, 228 journals were cited 1615 times. The articles were divided according to the corresponding department and Bradford coefficient was calculated to be 3.27. Therefore, two journals, namely the Journal of Endodontics and the Journal of Periodontology, were identified as core journals.

In total, 208 author names were recorded. The most frequent number of co-authors was four (33.8%), followed by five (18.9%), three (16.2%), two (12.2%), six (12.2%), seven (5.4%), and one (1.4%), respectively. The authors’ collective collaboration coefficient^5^ was found to be 73%.

Among all cited materials, accessibility in online, print, or both formats at the library of the Faculty of Dentistry were recorded for 76, 47, and 22 journals, respectively. From 103 journals not accessible, 73 journals were cited only once. Among 46 cited books, 32 books (seven as electronic/print and 25 as print) were accessible in the library and 14 were not.

The collaboration coefficient (73%) found in this research indicates good collaboration of the faculty members. Papers prepared from theses seem to have more ciations.^3^ Other scholarly work by researchers tend to have fewer select citations. Our results are in agreement with a previous study with the same subject matter.^1^ The core journals found in this research shared common titles with those of previous studies in Iran,^1 , 4^ probably reflecting the country-wide policy governing the accessibility of international scholarly journals. The findings support the journal subscription program of the library of the Faculty of Dentistry. However, further subscription to select titles including journals and textbooks can be provided.

**Table 1:** Frequency distribution (percent) of articles and citation resources in different departments

			Citations				
Department	Number of Articles	Mean number of references	Journal	Book	Internet	Thesis	Other
Operative Dentistry	27	23.55	602 (94.7)	33 (5.2)	—	1 (0.2)	—
Periodontics	17	23.76	388 (96)	16 (4)	—	—	—
Endodontics	14	27.64	361 (93.3)	20 (5.2)	—	3 (3.8)	3 (0.8)
Oral Medicine	6	11	54 (81.8)	9 (13.6)	3 (4.5)	—	—
Orthodontics	4	25	93 (93)	7 (7)	—	—	—
Oral Pathology	2	24.5	44 (89.8)	5 (10.2)	—	—	—
Oral and Maxillofacial Surgery	2	23	42 (91.3)	3 (6.5)	1 (2.2)	—	—
Pediatric Dentistry	1	20	18 (90)	2 (10)	—	—	—
Prosthodontics	1	15	12 (85.7)	2 (14.3)	—	—	—
Total	74	23.28	1614 (93.7)	97 (5.6)	4 (0.2)	4 (0.2)	3 (0.2)
